# Artificial Ovary for Young Female Breast Cancer Patients

**DOI:** 10.3389/fmed.2022.837022

**Published:** 2022-03-17

**Authors:** Jing Chen, Luz Angela Torres-de la Roche, Ulf D. Kahlert, Vladimir Isachenko, Hui Huang, Jörg Hennefründ, Xiaohong Yan, Qionghua Chen, Wenjie Shi, Youzhu Li

**Affiliations:** ^1^Reproductive Medicine Center, The First Affiliated Hospital of Xiamen University, Xiamen, China; ^2^University Hospital for Gynecology, Pius-Hospital, University Medicine Oldenburg, Oldenburg, Germany; ^3^Molecular and Experimental Surgery, University Clinic for General, Visceral and Vascular Surgery, University Medicine Magdeburg and Otto-von Guericke University, Magdeburg, Germany; ^4^Research Group for Reproductive Medicine and IVF Laboratory, Department of Obstetrics and Gynecology, Cologne University, Cologne, Germany; ^5^Reproductive Medicine Center, Women and Children's Hospital, Xiamen University, Xiamen, China

**Keywords:** breast cancer, artificial ovary, fertility preservation, cancer recurrence, follicle, stem cell

## Abstract

In recent decades, there has been increasing attention toward the quality of life of breast cancer (BC) survivors. Meeting the growing expectations of fertility preservation and the generation of biological offspring remains a great challenge for these patients. Conventional strategies for fertility preservation such as oocyte and embryo cryopreservation are not suitable for prepubertal cancer patients or in patients who need immediate cancer therapy. Ovarian tissue cryopreservation (OTC) before anticancer therapy and autotransplantation is an alternative option for these specific indications but has a risk of retransplantation malignant cells. An emerging strategy to resolve these issues is by constructing an artificial ovary combined with stem cells, which can support follicle proliferation and ensure sex hormone secretion. This promising technique can meet both demands of improving the quality of life and meanwhile fulfilling their expectation of biological offspring without the risk of cancer recurrence.

## Introduction

Breast cancer (BC) is the most widespread cancer in female worldwide (1, 2). The incidence of this cancer has remarkably increased since the 1970's, with the greatest boost in patients of reproductive age (3). Owing to the diagnostic and therapeutic advances, the mortality rate of women with BC is decreasing yearly (4). The long-term side effect of BC treatment is impaired or even loss of reproductive function. Premature ovarian failure (POF) may lead to insomnia, vasomotor symptoms, and osteoporosis and significantly disturbs mental function that determines the quality of life (5). Therefore, the greatest concern of these young survivors is to preserve and maintain their fertility (6).

## Risks on Ovarian Function During the Treatment of BC

First, surgical treatment may directly cause the loss of ovarian function. For BC with BRCA mutation carriers, bilateral salpingo-oophorectomy is recommended by the American College of Obstetricians and Gynecologists (ACOG) for reducing the risk of ovarian cancer (hereditary breast-ovarian cancer syndrome; HBOC) (7). Because BC with a positive BRCA mutation has a greater impact on ovarian reserve after chemotherapy treatment (8). Second, chemotherapy has genotoxic side effects. It has immediate and long-term side effects on ovarian function. A woman has finite primordial follicles (about one million) derived from the proliferation of primordial germ cells (PGCs) in their ovaries at birth, that is called a resting pool. But 85% of follicles in the resting pool are atresia before birth. Primordial follicles are recruited and activated to grow from the resting pool, most of them gradually to be atresia, and eventually, only one will ovulate during every menstrual cycle. When primordial follicles are <1,500 in the resting pool, this woman may quickly undergo menopause and lose ovarian function (9). The first effect of chemotherapy on the ovarian is immediate. It is cytotoxicity to dividing cells, which may directly kill growing follicles and induce POF. Chemotherapy may also induce inflammation and destruction of vascular and stroma, which is harmful to the growth of the follicle. However, as long as there are enough primordial follicles in the resting pool, this phenomenon can be reversible after the cessation of chemotherapy (10). Another relative side effect of chemotherapy is the long-term effect on the resting pool. The acute decrease in growing follicles, which leads to the reduction of sex steroid hormones and inhibin, may activate primordial follicles in the resting pool, enhance the rate of recruitment, accelerate the depletion of the reserve, and finally lead to POF (6).

The side effect caused by chemotherapy is dependence on the drug category used, the total dose given, and the duration of treatment. Alkylating agent is the strongest gonadotoxic drug that is widely used in BC chemotherapy. Cyclophosphamide is an alkylating agent, due to its similar DNA interstrand crosslinking agents, which can block the division of cells. Cyclophosphamide also may induce the expression of H2AX, which can break the double-strand DNA of follicles (11). Doxorubicin (adriamycin) can cross the physiological barrier of the follicle, directly acts on the DNA of oocytes, and induces cell apoptosis. Moreover, follicles in the germinal vesicle (GV) stage were more vulnerable to this toxic effect (12). Antimetabolite cytotoxic drugs often used for BC therapy, for example, fluorouracil and epirubicin, which are specific to the S-phase of the cell cycle (DNA synthesis), and have a high risk of ovarian toxicity (6).

Thirdly, radiotherapy has detrimental effects on ovarian function. Follicle is strongly sensitive to ionizing radiation. It can directly or indirectly impair ovarian function. When radiation targeted the pelvis, abdomen, or total body, it will directly impair fertility. When ovary is put away from the radiation range, some escaping radiation will be scattered and will indirectly impair fertility (10). The frequency of POF after radiotherapy is related to the used dose of radiation. Whole irradiation doses at 3–5 Gy, 60% of the follicles are destroyed; with irradiation at doses of 5 Gy, 100% of the follicles are destroyed (10). When at doses of 20 Gy, 71% of women during childhood failed to enter puberty (13).

Finally, risk of POF caused by chemotherapy is dependence on the female's age at breast cancer treatment. More than 80% of childhood cancer survivors have long-term survival into adulthood. These survivors have a 1.48-fold higher risk of POF than their siblings (14). Anti-Müllerian hormone (AMH) was detected falling rapidly in both prepubertal and pubertal girls undergoing cancer therapy (15). Another risk of POF is age-related resting pool decline on the number of primordial follicles. Because female cancer survivors are often advised to postpone pregnancy due to the risk of recurrence. For example, BC survivors with hormone receptor-positive are advised to delay pregnancy for up to 10 years after chemotherapy (6).

## Strategies for Fertility Preservation

### Medical Gonadoprotection

Medical gonadoprotection through ovarian suppression using GnRHa (gonadotropin-releasing hormone agonists) can inhibit the maturation of oocyte. Its molecular structure is similar to native GnRH but has a higher affinity to receptors. In the beginning, it can flare up the ovarian hormone secretion (LH, FSH). After 7 days, the reduction of functional GnRH receptor may decrease the release of LH and FSH, which leads to the decrease of primordial follicles' recruitment and development. So, GnRHa administer should start 7 days before chemotherapy and continues until the end of therapy (16). The decrease of ovarian hormone secretion can downregulate blood supply to the *utero*-ovarian, thereby reducing the drug entering the ovaries. The use of GnRH analogs to protect ovarian function during chemotherapy treatment is controversial (17, 18). It can interfere with anticancer therapy (19), and it also may induce reversible menopausal symptoms (20). An analysis by Lambertini et al. showed a higher pregnancy rate in women undergoing chemotherapy combined with GnRHa. But this result is still not ideal, the pregnancy rate in the chemotherapy-GnRHa group is only 9.2%, whereas in the chemotherapy-alone group is 5.5% (21). Hence, for patients with BC undergoing fertility preservation, GnRHa can only be used as an additional treatment to oocyte–embryo cryopreservation, but it cannot replace it.

### Oocyte–Embryo Cryopreservation in Patients With BC

Although young women with BC face challenges in fertility, there are still many data showing that patients with a history of BC successfully conceive and do not relapse, even in patients with BC with estrogen receptor (ER)-positive (22) or germline BRCA mutations (23). Fertility restoration by oocytes and embryos cryopreservation should be highlighted for young BC women before anticancer therapy. Many studies have shown that the storage duration of cryopreservation had no negative effects on clinical outcomes (24, 25). Additionally, the pregnancy rate in frozen-thawed embryo transfer is even higher than fresh embryo transfer (26). It is established that fertility preservation and reproducible method can be safely and efficiently without being interfered with by anticancer treatments (27, 28). But it has some limitations. For embryo cryopreservation, it requires sperm to fertilize, which is difficult and unacceptable for single BC women. Oocyte and embryo cryopreservation need ovarian stimulation to retrieve mature oocytes, which may be considered contraindicated for patients with BC due to its high levels of estradiol generated by stimulation. Ovarian stimulation is also not feasible for patients with BC in childhood or prepubertal girls. In addition, the ovarian stimulation cycle usually takes 7–14 days, and there is a risk of ovarian hyperstimulation. If ovarian hyperstimulation occurs, it will take another 7–14 days to recover. These may delay the timing of anticancer treatment, which is not suitable for patients with who need immediate anticancer treatment (29).

### Ovarian Tissue Cryopreservation

Ovarian tissue cryopreservation (OTC) before anticancer therapy and autotransplantation after healed is an emerging and successful method for young BC females that has produced more than 180 babies (16). OTC is a surgical method that can be carried out at any stage of BC, it not only can preserve fertility, but also restore endocrine function, produce a natural level of hormones, and have been considered as an established strategy for young patients with BC in many countries (30). OTC does not need ovarian stimulation nor require sperm and can be performed in aged 0–40 years, especially for children without delay the timing for anticancer therapy. Gellert et al. review data about 328 women who underwent autologous retransplantation of ovarian tissue, nearly 95% restored hormonal function, 72% recovered fertility function, and 40% were pregnant (31). Pacheco et al. also recorded a 65% of endocrine renovation and produced a 37% of pregnancy rate in patients with OTC and autotransplantation (32). The pregnancy and birth by OTC are increasing steadily and are exceeded 200 of live birth (33).

The risk of reimplanting residual neoplastic cells in ovarian tissue is a major safety issue (34). Ovary contaminated by BC is not uncommon, and nearly 13–47% of BCs have ovarian metastases (35). These cases were asymptomatic and often diagnosed accidentally based on autopsy or ovarian surgery, which suggests that the incidence of ovarian metastasis was underestimated (36). Both invasive lobular carcinoma and invasive ductal carcinoma in BC were reported about BC cells metastasizing to the ovaries (35). Besides, ER-positive BC and BC with axillary lymph node metastasis are positively correlated with ovarian metastasis (36). Furthermore, BC at stages III–IV and inflammatory BC are more likely to have ovarian metastasis (37). Hence, the OTC strategy for fertility preservation in young BC females should be aware and handled with caution due to the higher risks of ovarian metastasis and cancer recurrence. In these patients, the emerging technology of artificial ovary which can be an ideal alternative strategy to preserve and restore fertility should be emphasized.

### Artificial Ovary

Considering the risk of reimplanting the metastasis BC cell by autotransplant OTC, artificial ovary as a promising fertility-restoring alternative approach has been investigated by many research groups from worldwide (38, 39). Although this strategy remains challenging for clinical use, promising results have been reported in animal models. Laronda et al. isolated follicles from cryopreserved human ovarian tissues to form an artificial ovary and transplanted them into ovariectomized adult mice. A number of 6 out of 7 ovariectomized mice with artificial ovary implanted had recovered hormone cycle in 4 weeks (38). Kniazeva et al. extracted follicles from young female mice and encapsulated them into an artificial ovary, mice for subsequent transplantation and mated. Nearly 33% of female mice deliver offspring (39). The main target function of the artificial ovary is to prevent reimplantation of malignant BC cells and mimic the function of the ovary. It can offer BC women opportunities to have their genetic offspring and recover endocrine function without cyclic hormone replacement therapy.

### Management for Creating a Safe Artificial Ovary

Breast cancer cells spread through lymphatic and blood vessels to invasive the ovaries and colonize (37). Follicles in ovary are surrounded by a basement membrane as a protective barrier to avoid direct contact with blood vessels, capillaries, and white blood cells which can protect follicles from being contaminated by malignant BC cells (34). Follicles also is a functional unit in ovary secreting hormones and regulating the menstrual cycle. Hence, preserving follicles is a fundamental part of safely preserving reproductive function. Fortunately, primordial follicles in resting pool population in the outer cortical region of ovary and these stages of follicles are most stable for cryopreservation due to the absence of spindle, zona pellucida, and the smallest of follicular size (40). Theoretically, a small biopsy of ovarian cortex is enough for cryopreservation, because there are numerous follicles in the resting pool in the cortex. But to increase the success rate of fertility preservation, 1/2–2/3 of the cortex from one ovary should be cryopreserved in BC cases (41). Therefore, ovarian cortex cryopreservation is stable, and its isolation for retrieval follicles is a safe and well-preserved fertility function without metastasis by BC malignant cells.

To retrieve the most quality and quantity of follicles, several follicle isolation methods have been proposed and tested due to the fibrous structure of ovarian cortex. The mechanical isolation method was first used for follicle isolation and is the best method to preserve the morphology of follicles. It can generate follicles with intact basement membrane and less granulosa cell loss. But mechanical isolation is a laborious process that takes a long time, only a small part of the follicles can be isolated, and most of them remain in the tissues (42). Enzymatic digestion is an alternative approach including collagenase, Liberase, and TDE enzyme can isolate the greatest quantity of follicles, but most of them were granulosa cell lost or membrane damaged because the enzyme can digest the extracellular matrix and degrade the basement membrane (43, 44). The most effective method is the combination of mechanical isolation and enzymatic digestion yielding high quality and quantity of follicles (45, 46). Chiti et al. use a modified protocol by filtering the digestion solution every 30 min. After filtering, the isolated follicles were picked up and the remaining fragments were redigested until completely digested. This modified protocol can fully digest all types of ovarian tissue with a good preserve of isolated follicles from prolonged exposure to enzyme solution which may toxic and damage for follicles (46).

Ensuring the safety of the follicular isolation procedure without metastasis by malignant cells is a crucial step for the artificial ovary because both follicles and BC cells are involved in the digested solution. During the follicular retrieval process, follicles may also contaminate by malignant cells and replant into the artificial ovary. Soares et al. transplanted 100 leukemic cells inside an artificial ovary and grafted it into mice, none showed any sign of leukemia after 20 weeks, with reassurance by IHC and PCR method which showed all negative in the recovered ovary. It appears that grafting 100 leukemic cells is insufficient to induce leukemia (47). Meanwhile, further verified through 3 washes of follicles can effectively eliminate malignant cells without affecting the viability of follicles (48), and repeated experiments by multicolor flow cytometry (MFC) also confirmed this result (49).

### Management for Creating a Functional Artificial Ovary

**Figure 1:**
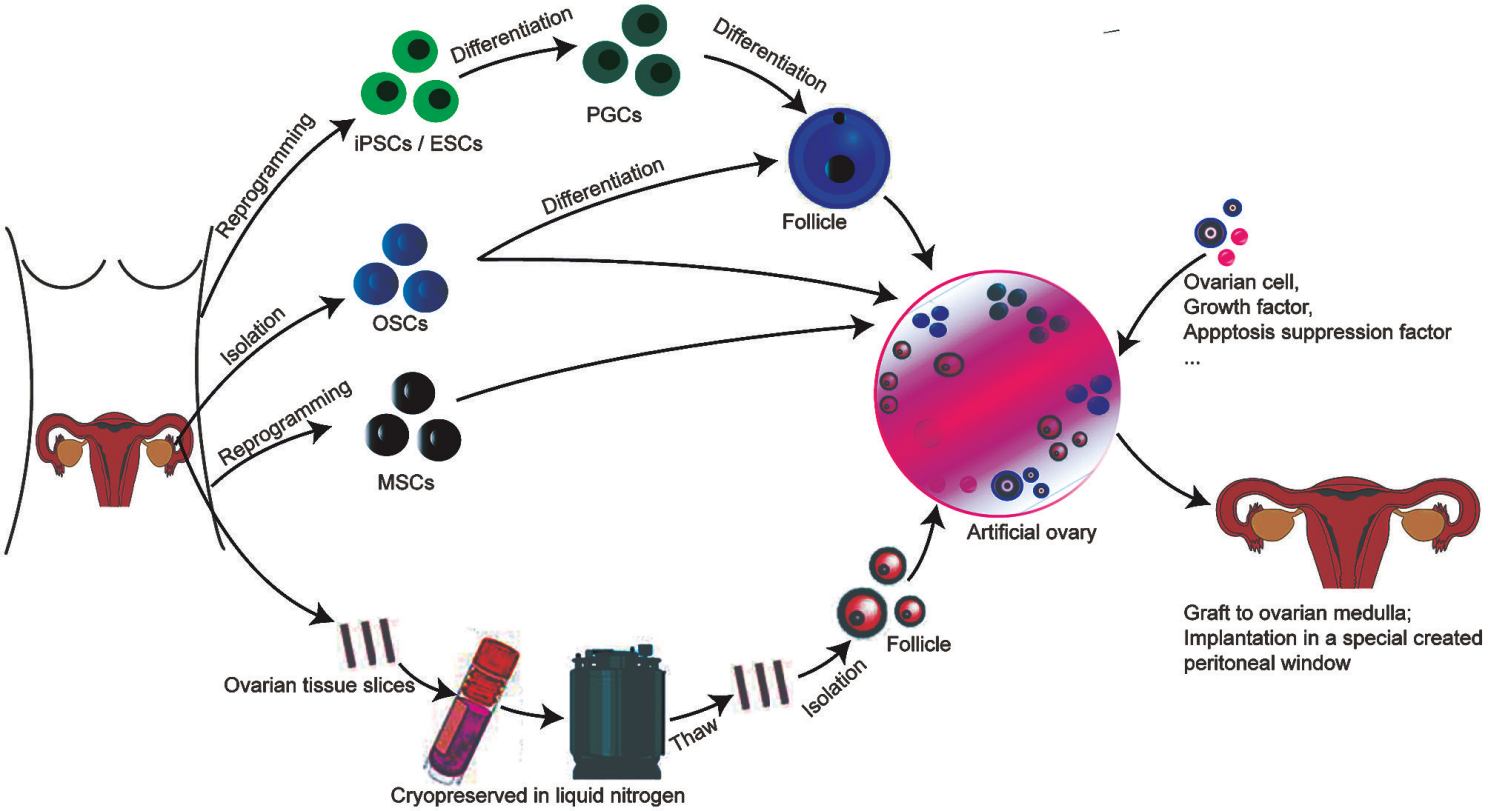
Protocol for constructing an artificial ovary combined with stem cells for patients with BC to preserve fertility and restore endocrine function. (I) If the patient is prepubertal or requires immediate chemotherapy with a potential risk of transmitting malignant cells, ovarian tissue slices are removed and long-term cryopreserved in liquid nitrogen. After thawing, follicles can be isolated from ovarian tissue and then embedded inside a scaffold, we called it “artificial ovary.” (II) Patient-specific induced stem cells, such as PGCs, ESCs, and OSCs, can be differentiated to form follicles and embedded into an artificial ovary. OSCs and MSCs can also be directly placed inside the artificial ovary without differentiation. (III) Other additives can also be added into the artificial ovary, such as ovarian cells, growth factors (VEGF, bFGF), and apoptosis suppression factor (S1P). Finally, this transplantable and functional artificial ovary can be grafted to the ovarian medulla or implantation in a specially created peritoneal window.

Folliculogenesis is a complex regulated by interaction among follicles, ovarian cells, and environmental (50). Mimicking the natural environment of the follicle to support follicle survival and development is vital for creating a functional artificial ovary. Isolated primordial follicles that are fragile need a scaffold to support three-dimensional structure (51). Interestingly, isolated follicles cultured in alginate scaffold together with theca and stromal cells had higher survival and development rates, which indicate that extracellular scaffold together with other cells as their native tissue microenvironment benefits follicle growth. Growing follicles with multilaminar structures can grow up in such tissue engineering scaffolds (52). Hence, for constructing an artificial ovary, we need a suitable scaffold that can maintain follicular three-dimensional structure and with other cells or factors which could allow follicle-cell-matrix dynamic signal communications to lead to an ovary-like environment (Figure 1).

### Design a Suitable Scaffold for Artificial Ovary

The ultimate goal of artificial ovaries is to be retransplanted into the human body, so its ingredients must be biosafety and tolerable by the human body. The diameter of follicles' folliculogenesis from the primordial stage is 19–30 μm to the mature stage 100–110 μm, so this scaffold should be degradable for follicle growth and migration. Additionally, it also should be high-temperature resistance due to the human body temperature (34). In addition, follicles need signal communication with cells and their environment, and this 3D matrix should be high penetrated to allow the diffusion of nutrients and signal pass in and out the scaffold. Isolated follicles were fragile but it is stable while embedded in a 3D scaffold and is convenient and safe to manipulate and handle without disrupting the follicular structure (53). Overall, this 3D scaffold should be (i) biosafety and tolerable by the human body, (ii) resistant to the human body temperature, (iii) liable for cell adhesion, proliferation, and differentiation, and (iv) allow the dissemination of nutrients, growth factors, and oxygen. Tissue engineering using biomaterial supporting artificial ovary varies from natural (collagen, plasma clot, alginate, fibrin, decellularized tissues, etc.) to synthetic polymer (polyethylene glycol, 3D printing ovary, etc.) with promising and encouraging outcomes conducted in animal research models. Natural polymers are not rigid enough to support the mechanical structure, but it is superior for cell adhesion, migration, and signal communication. Synthetic polymers are superior for supporting mechanical properties when grafted in the human body, but they lack molecules for cell adhesion which is not conducive for nutrient exchange and signal crosstalk (54).

Collagen and plasma clot was the first natural scaffolds used to embedded isolated primordial follicles. Telfe et al. isolated follicles from mice and cultured them in a collagen matrix for 5 days and then grafted them to ovariectomized mice. Follicle can develop to a mature stage and can produce hormones enough to support vaginal opening and cornification of vaginal epithelium in ovariectomized mice after 5 days of transplant, and blood vessels also appear in the grafted gel. Mature follicles that are extracted from this grafted gel can be harvested and finally resulted in the embryo through *in vitro* fertilization (55). In the same year, Gosden et al. isolated primordial follicles from infant mice and culture in a plasma clot and then transferred them back into a vacant periovarian capsule which was immediately formed after ovariectomy. All stages of follicular maturation can be seen in the grafted clot, eleven of fifteen mice were pregnancies, and 2 mice produced offspring (56). Dolmans et al. isolated human primordial follicles, embedded in plasma clot, and xenotransplanted to immunodeficient mice. Secondary stage and antral follicles can be found in clots after 5 months of transplanted, but plasma clots were degraded quickly leading to a large number of follicle losses (57, 58). Hence, the contraction and deformation characteristics of collagen or plasma clot scaffolds are difficult to manipulate, and follicles are easy to lose which has limited their application to load-bearing tissues in the human body (34, 54).

Alginate is a polysaccharide-based natural polymer derived from algae, and the rigidity of alginate can avoid structure from being degraded. Rios et al. encapsulated isolated follicles from mice into alginate matrix and transplanted them back into ovariectomy mice. Many follicles can develop into antral follicles and even mature follicles which can be successfully fertilized by intracytoplasmic sperm injection (ICSI) (59). It is reported that embedded isolated human primordial follicles in alginate gel and culture *in vitro* for 8 days, follicles can develop, and some of them can reach the preantral stage (60). But when the culture *in vitro* for a longer time (>30 days), follicles grow to the antral stage, but many of them were degenerated and stopped growing after further culture (61), since human follicles are larger than mouse follicles, alginate is rigid and cannot be degraded without alginate lyase, which can limit the further growth of follicles and also not conducive for vascularization (62).

Fibrin is another natural polymer to replace plasm colt, fibrinogen and thrombin are the main components of fibrin, and their concentration determines the porosity and hardness of fibrin. Fibrin has high bioadhesion with minimal inflammation after being grafted into the human body and has been widely used for tissue engineering. Paulini et al. isolated human primordial follicles encapsulate in fibrin gel and xenografted in mice, and many of the follicles can grow into the second stage after 7 days of xenografted (63). Long-term (21 days) culture in fibrin gel of isolated mice primordial follicles can also be developed into the antral stage, and hormone levels can be detected in the mice (64). But fibrin has a higher degradation rate in the human body, due to the inherent plasmin and other inhibitors in the human body, follicles will lose the support of the architecture after the degradation. Fortunately, the degradation of fibrin is safe from toxic and can be naturally cleared by the human body (65). In the natural ovary, the outer cortex is more solid whereas the medulla is soft which can allow follicles to migrate from solid cortex to soft medulla (66). An interpenetrating network composed of fibrin-alginate was investigated for embedding mice secondly follicle for short-term culture and produced a higher meiotic rate of oocyte than alginate or fibrin along (67). Longer-term (30 days) cultures of isolated caprine follicles in a fibrin-alginate matrix have a higher maturation rate than alginate only (68). We can infer that a partially degraded fibrin-alginate matrix is beneficial for follicle survival and proliferation with adjustable rigidity and degradability.

Decellularized ovarian extracellular matrix is another natural matrix, obtained by removing the cellular components from the natural ovary, which can highly mimic the natural ovary *in vivo* allow cells to adhere and grow. Decellularized tissue has been tested in the liver (69), lung (70), and heart (71). Nevertheless, xenotransplantation may induce the immune reaction in the human body and should be paying more attention to further clinical application. Laronda et al. seeded isolated mice follicles into decellularized bovine ovary scaffolds and grafted them to normal ovariectomy mice with normal immune function. After 2 weeks of transplantation, an antral follicle can be discovered in grafted scaffold (38). Isolated mice follicles were also cultured in a decellularized porcine ovary and regrafted back natural pregnancy, and healthy offspring was generated in the POF mouse model after 100 days of graft (72). Hassanpour et al. decellularized the human ovary embedded with isolated rat follicles and grated back into a rat. Hormone and primordial or primary follicle-like structures were detected in this suitable cytocompatibility scaffold after 4 weeks of surgery (73). Pors et al. also successfully embedded isolated human follicles in a decellularized human scaffold and grated them back into a rat for 3 weeks (74). But xenogeneic scaffolds may induce a high risk of the immune response and also may induce some diseases, for example, viruses, or cell residues from the donor (75).

Synthetic polymer has its advantage compared to the natural polymer. It can tailor according to the different hardness of the natural ovary and meet the different clinical requirements (76). Polyethylene glycol (PEG) is widely used for engineering, and oxygen and carbon are the main components of PEG. Kim et al. use PEG hydrogels for embedding isolated mice follicles and grafted into ovariectomized mice, and each stage of follicles and corpora lutea can be discovered in scaffold after 30 days of grafting. Hormone levels improve significantly after 60 days of graft, and functioning blood vessels can also be detected in the scaffold (77). However, the degradation of PEG hydrogels is toxic, and the degradation products can easily cause immune response (78).

3D bioprinting can precisely adjust the pore size and thickness of the stent and can also control the rigid and other properties of the scaffold to meet the clinical needs. It can create the scaffold layer-by-layer to generate a tissue mimic structure (79). Laronda et al. use gelatin as 3D ink to print a scaffold crosslink with 250 μm diameter of the strut, 350 μm diameter of the pore. After seeding isolated mice follicles in 3D printed scaffold, the scaffold was grafted into ovariectomized mice and became vascularized after 7 days of implantation without additional exogenous angiogenic factors. Mature follicles can be found after 3 weeks of implantation, after 10 weeks, these grafted mice were mated, and each recipient mice have one or two litters (80). Other seeding isolated porcine follicles in a scaffold composed of gelatin together with poly(epsilon-caprolactone) (PCL), to construct a structure with 300 μm of pore size and 1 μm diameter of struts. After 10 days of *in vitro* culture, the follicle can adhere well to the stent with good development and a high survival rate (81).

## Additives for Transplantable and Functional Artificial Ovary

### Stem Cells for Generating New Oocytes in Artificial Ovary

Stem cell is an alternative source and promising strategy for constructing an artificial ovary with regenerative function. Pluripotent stem cells have self-renewal and differentiation functions. Additionally, a functional oocyte in mammals needs multiple steps of generation from a germ cell. Embryonic stem cells (ESCs) and induced pluripotent stem cells (iPSCs) can be induced to primordial germ cells (PGCs) or primordial germ cell-like cells (PGCLCs), in turn, differentiated to the oocyte. Female oogonial stem cells (OSCs) originate from very small embryonic-like (VSEL) stem cells that exist in the ovary and have the ability of oogenesis without inducing differentiation. In oogenesis, these differentiations becoming primary oocytes were also regulated by the environment in the artificial ovary on transplantation into the body.

#### Induced Pluripotent Stem Cells and Embryonic Stem Cells

Hayashi et al. induced iPSCs to perform PGCLCs and transplanted the PGCLCs into mice seminiferous tubules. After 10 weeks of transplantation, spermatogenesis was exhibited and can form an embryo followed by ICSI and successfully resulted in offspring (82). Subsequently, they derived female ESCs like ESCs and iPSCs to perform epiblast-like cells (EpiLCs) and further induce it to PGCLCs, later coculture it with embryonic gonadal somatic cells to form ovary *in vitro* and then transfer this artificial ovary to mice for oogenesis, follicles at GV stage were detected in 32 days after transplantation, and mature oocyte can be isolated at 53 days after transplantation, which can be well fertilized and generate offspring (83). Hence, ESCs–iPSCs can be a promising source for generating new oocytes for artificial ovaries. Despite these encouraging results, induced iPSCs may have a risk of mitochondrial mutations, and we should pay more attention to pathogenic mitochondrial DNA modifications after transplanting it *in vivo* (84).

Female oogonial stem cells that are extracted on the surface of the ovary can generate primordial follicles. Many studies have confirmed the existence of OSCs in the human ovarian cortex (85, 86). Compared to ESCs and iPSCs, OSCs initially arise from VSEL stem cells and have the ability of oogenesis without inducing differentiation (87). White et al. obtained OSCs from human ovaries and *in vitro* manipulation, and oocytes were generated after 2 weeks of xenotransplantation in mice (88). Zou et al. isolated OSCs from mice ovary and transplanted them into the ovary of POF mice which was induced by chemotherapy. Oocytes were detected in the recipient ovary after 8 weeks of transplantation, and offspring was generated after long-term transplanted (more than 15 weeks) (89). Another repeated study obtained OSCs from mice ovaries and grafted them into adult mouse intraovarian. Follicles can be successfully generated, and 15% of offspring was delivered after natural mating in these grafted mice (90).

Mesenchymal stem–stromal cells (MSCs) that were obtained from the bone marrow have self-renewal potential without pluripotent function. It also can be retrieved from menstrual blood, cord blood, and adipose tissue as a paracrine provider to support stem cell growth and differentiation (91). Although it cannot directly differentiate to the oocyte, transplantation MSCs can secrete cytokines, signal, and growth factors to promote stem cells such as ESCs, iPSCs, and OSCs in artificial ovary differentiate into oocytes (92). MSCs can also simultaneously support nutrition and immune regulation for the ovary (93). It was reported that transplantation of MSCs can provide nearly 109 cytokines in the ovary to help recover follicles in POF patients (94). Wang et al. grafted green fluorescence protein (GFP, Genechem, China)-positive MSCs to the ovary and found that they gather in the interstitium instead of follicles in the grafted ovary (95). The umbilical cord (UC) is the most promising source of MSCs in humans (UC-MSCs) due to its low oncogenicity and rapid self-renewal. Yang et al. embedded human UC-MSCs into a collagen matrix and then transplanted it into POF mice. After 2 weeks of transplantation, hormone levels and follicles' number have risen significantly, and granulosa cell proliferation and ovarian angiogenesis were detected in the graft (96). Transplantation of MSCs can boost ovarian function and improve the success rate and outcome of the artificial ovary *in vivo*.

### Ovarian Cell for Functional Artificial Ovary

An ovarian cell can support angiogenesis, and signal transduction of the artificial ovary is fundamental for follicle proliferation and maturation (97). Ovarian cells can secrete factors that can regulate the transformation of primordial follicles into primary follicles and simulate the microenvironment for follicle growth and survival proliferation (98). There is a positive correlation between the number of human ovarian stromal cells and endothelial cells, the area of angiogenesis, and the survival of follicles in the artificial ovary after transplantation *in vivo* (99). Dath et al. embedded isolated human stromal and endothelial cells together with follicles into plasma clots and xenografting in mice ovary. Fully vascularized stromal structure and higher scaffold degradation were detected in graft after short-term xenograft (100). Additionally, the best source for ovarian cells comes from fresh medulla part in the ovary after cancer remission, and this strategy not only can reduce the risk of reintroducing the malignant to the body, but it also can avoid the damaging effect of cryopreservation to ovarian cells. Because ovarian cells are sensitive to cryopreservation, chemotherapy has less effect on ovarian cells (97). Another source for the ovarian cell is the stem cell. Park et al. isolated stem cells from mice skin, and we can call it skin-derived stem cells (SSCs), induced SSCs differentiation to ovarian-cell-like cells, embedded in Matrigel scaffold, and then transplanted into ovariectomized mice. Estrus cycles were recovered, and follicles and blood vessels were found in the transplants after 8 weeks of transplantation (101).

### Factors for Supporting the Artificial Ovary

Growth factors such as vascular endothelial growth factor (VEGF) and basic fibroblast growth factor (bFGF) can promote angiogenesis and decrease apoptosis for artificial ovary *in vivo*. Shikanov et al. embedded ovarian tissue together with VEGF in fibrin gel and grafted it back into bilateral ovariectomy mice. After 2 weeks of transplantation, a gel containing VEGF has two times as many survival follicles and blood vessels as the control group (102). Another study also encapsulated ovarian tissue together with bFGF in fibrin gel and then grafted it under the skin of mice. After 7 days of transplantation, the bFGF group has higher follicle survival and proliferation rate, lower follicle and ovarian cell apoptosis rate, and higher angiogenesis rate compared to the non-bFGF group (103).

Apoptosis suppression factor sphingosine-1-phosphate (S1P) is one of the apoptosis suppression factors that can induce cell survival and proliferation. It is a signaling sphingolipid that can act as an intracellular second messenger and extracellular ligand for G protein-coupled receptors. It also can regulate angiogenesis and vascular stability (104). Soleimani et al. reported that xenograft of human ovarian into severe combined immunodeficient (SCID) mice together with S1P. After 10 days of transplantation, vascular density, angiogenic, and proliferation of ovarian cells were increased significantly in graft, with lower follicle apoptotic compared to the control group (104). Another research embedded follicles together with S1P and VEGF into fibrin scaffold and generated two times as many primordial follicles, blood vessels, and offspring compared to the control group (105).

## Conclusion and Future Endeavors

The number of young women who are diagnosed with BC has risen continuously in recent years. Simultaneously, the development of modern therapeutic significantly improved the survival rates and prolong the life expectancy. Hence, fertility preservation turned out to be an urgent request for young females before gonadotoxic therapy. Artificial ovary combined with stem cell can mimic natural ovary as a promising strategy for patients with BC that meets the needs of recover fertility and restore gonadal hormone function without reintroducing the malignant cells and delaying their cancer therapy.

As a transplantable in the human body, the scaffold of artificial ovary should allow follicle survival and proliferation, facilitate the formation of blood vessels and stroma *in vivo*, and should be safe for the human body. Although animal research has generated few offspring on artificial ovaries, more experiments and animal studies should be tested to search for a suitable scaffold for transplantable artificial ovaries. Due to the finite source of follicles, stem cells as an alternative to female gametes bring great hope for future clinical implementation. Yet, stem cell therapy is still in the research stage and is insufficient for clinical use. More research is needed to verify and test for fill gaps that may lead to clinical benefits in the future.

## Author Contributions

JC wrote the manuscript and figures. JC, LR, and YL reviewed the literature. QC, XY, and WS conceived the framework of this review article, provided insights, and edited the manuscript. VI and HH revised and polished the manuscript. UK and JH edited the grammar and revised the manuscript. YL polished the final manuscript and figure. All authors contributed to the article and approved the submitted version.

## Funding

This work was supported by the Key Medical and Health Program of Xiamen (no. 3502Z 20209001).

## Conflict of Interest

The authors declare that the research was conducted in the absence of any commercial or financial relationships that could be construed as a potential conflict of interest.

## Publisher's Note

All claims expressed in this article are solely those of the authors and do not necessarily represent those of their affiliated organizations, or those of the publisher, the editors and the reviewers. Any product that may be evaluated in this article, or claim that may be made by its manufacturer, is not guaranteed or endorsed by the publisher.

## Abbreviations

POF: premature ovarian failure
BC: breast cancer
ER: Estrogen receptor
ACOG: American College of Obstetricians and Gynecologists
HBOC: hereditary breast-ovarian cancer syndrome
GV: germinal vesicle
GnRHa: gonadotropin-releasing hormone agonists
OTC: ovarian tissue cryopreservation
COH: controlled ovarian hyperstimulation
3D: three dimensional
ICSI: intracytoplasmic sperm injection
ESCs: embryonic stem cells
iPSCs: induced pluripotent stem cells
PGCs: primordial germ cells
PGCLCs: primordial germ cell-like cells
OSCs: oogonial stem cells
VSEL: very small embryonic-like
SSCs: skin-derived stem cells
bFGF: basic fibroblast growth factor
S1P: sphingosine−1-phosphate
SCID: severe combined immunodeficient.
